# Serial memory for landmarks encountered during route navigation

**DOI:** 10.1177/17470218211020745

**Published:** 2021-05-25

**Authors:** Christopher Hilton, Jan Wiener, Andrew Johnson

**Affiliations:** 1 Psychology Department and Ageing & Dementia Research Centre, Bournemouth University, Bournemouth, UK; 2 Biological Psychology and Neuroergonomics, Berlin Institute of Technology, Berlin, Germany

**Keywords:** Serial memory, sequence learning, navigation, route learning, ageing

## Abstract

The present study demonstrates similarities between route learning and classical tests of serial order memory. Here, we investigated serial memory for landmarks in a route learning task, in younger and older adults. We analysed data from a route learning task with 12 landmarks. Participants (88 younger and 77 older) learned a route using either a Fixed Learning (3 exposures to the route) or Flexible Learning (repeated exposures until successful navigation was achieved) procedure. Following route learning, participants completed Immediate Free Recall (IFR) and Free Reconstruction of Order (Free RoO) of the landmarks. We show clear acquisition of sequence memory for landmarks for both age groups, with Free RoO producing a bowed serial position curve. IFR produced recency effects but no primacy effects in fixed learning, with recency reduced following flexible learning for both age groups. Younger adults displayed a primacy bias for the first item recalled in both learning conditions, as did the older adults in the flexible learning condition. In contrast, older adults displayed a recency bias in the fixed learning condition. Evidence of contiguity in IFR was present only for younger adults in the flexible learning condition. Findings are broadly consistent with results from typical short-term list learning procedures and support the universality of sequence learning effects, which we demonstrate are generalisable to a navigation context.

## Introduction

Recall of a sequence is typically characterised by a bowed serial position function in which a memory advantage is observed for the first (primacy) and last (recency) items in the sequence. The ubiquity of this serial position function has been hypothesised to represent a benchmark of short-term/working memory (Oberauer et al., 2018), and more generally, an underlying feature of memory (e.g., Surprenant & Neath, 2009). The present study examines sequence knowledge in a route learning paradigm and provides a detailed analysis for some of the benchmark findings observed in sequence memory. Specifically, we examine whether such established findings can be generalised to the learning of landmarks during navigation, despite the different characteristics of the two tasks.

Landmarks are objects or distinctive features in the environment which are used as cues for action during route navigation (Foo et al., 2005; Waller & Lippa, 2007). They are a key component in the development of spatial knowledge (Chrastil, 2013; Siegel & White, 1975). Indeed, recognition memory is greater for objects used as landmarks (Janzen, 2006), which yield selective recruitment of the parahippocampal gyrus (Janzen & van Turennout, 2004; Janzen & Weststeijn, 2007). Landmarks along a route are known to be linked to other proximal locations for purposes such as error monitoring, response preparation, or resolving ambiguous situations (Schinazi & Epstein, 2010; Strickrodt et al., 2015; Trullier et al., 1997). As such, understanding the role of serial position memory in landmark learning is useful for conceptualising how routes are represented in memory. Indeed, the use of visual cues has been described as a serial learning task embedded within a navigation task (Caplan et al., 2001).

The established bowed serial position function has already been reported in some navigation studies. In retracing a route around a university campus, children (8- and 12-year olds) exhibited strong recency and some primacy in recalling the correct direction at each intersection (Cornell et al., 1996; see also Meilinger et al., 2016, for similar effects within a virtual environment). In addition to accuracy at intersection decision points, primacy and recency have also been shown for the vividness of memories for landmarks encountered along a route. Helstrup and Magnussen (2001) instructed participants to remember landmarks positioned along a route to a frequently visited vacation destination, with participants self-reporting more vivid memories for landmarks positioned towards the start and end of the route. Finally, Stawarczyk and D’Argembeau (2019) instructed participants to recall thoughts experienced during a 25 min walk. Memory was better for thoughts encountered at the start and end of the walk, with these thoughts arguably functioning as internal landmarks encountered during the walk. The finding that free recall of those thoughts exhibited both primacy and recency as well as asymmetric temporal contiguity effects (i.e., a tendency to recall items in forward order despite ordered recall not being a task requirement) are consistent with conventional Immediate Free Recall (IFR) tasks (Bhatarah et al., 2008; Cortis et al., 2015; Spurgeon et al., 2014).

The fact that naturalistic wayfinding tasks exhibit canonical serial position effects is unsurprising given the ubiquity of bowed functions in list recall. Indeed, primacy and recency effects are not confined to episodic memory and are generalisable to the recall of semantic information. For example, when participants are instructed to order a list of category members on a given dimension, such as US presidents (Neath, 2010; Roediger & DeSoto, 2014), hymn verses (Maylor, 2002), age of actors (Kelley et al., 2015) and books in a series (Kelley et al., 2013), primacy and recency effects are evident. These results are consistent with the proposal that primacy and recency are general features of lists due to the first and last (i.e., boundary) items being more distinctive by virtue of having positional competitors on only one side (Kelley et al., 2015; Neath et al., 2016). Traditional dual-store accounts of serial position functions (e.g., Atkinson & Shiffrin, 1971; Murdock, 1967), where recency is a product of storage within a highly fragile short-term store, are inadequate in accounting for these effects. As an alternative to separate short-term and long-term stores, some researchers have argued for general principles of sequence memory that can be applied across differing timescales (e.g., Brown et al., 2007; Tan & Ward, 2000).

The current study presents analyses of a dataset that was collected in a previous study (Hilton et al., 2021), which contained measures of landmark recall and sequence memory, to expand our understanding of how typical sequence memory effects transfer to a navigation task. Hilton et al. (2021) conducted three experiments to examine the route learning capabilities of younger and older adults. Participants were presented with a to-be-remembered route comprising 12 decision-points, each containing a unique landmark. In Experiment 1, participants received three exposures to the route and in Experiments 2 and 3, they were exposed to the route repeatedly until they achieved at least 90% accuracy for the decision points (i.e., for the three-alternative forced choice decision of traversing right, left, or straight ahead). At test, participants performed (1) IFR of all the landmarks from the route, (2) an Associative Cue Task in which they were shown the 12 landmarks in a randomised order and were required to indicate the direction of travel (right, left, and straight), (3) Free Reconstruction of Order (Free RoO) wherein participants were given images of the landmarks and required to position them in the order they were encountered along the routes, and (4) the Missing Landmark Task, during which participants had to recall directions at intersections with the landmarks removed.

Hilton et al. (2021) established that while IFR of landmarks was comparable between age groups in all experiments, older adults performed worse on the Associative Cue and free RoO tasks when limited to only three exposures to the route. When rate of learning was controlled for in Experiments 2 and 3, the age-related performance deficit on the Associative Cue Task was attenuated, but performance deficits on free RoO and the Missing Landmark Task remained. These patterns of performance reflected different route representations formed by the participants in each age group. Hilton et al. (2021) suggested that older adults amended their learning strategies to obtain task-essential knowledge in a piecemeal manner, resulting in longer learning times and overall declines in the quality of spatial knowledge. Younger adults, however, appeared to engage in more parallel acquisition of different knowledge types resulting in a richer representation of the environment.

The present study is concerned with the different question of how sequence memory from route navigation reflects general sequence learning processes. No such insights were presented in Hilton et al. (2021), who analysed the IFR and free RoO data only in terms of overall performance (i.e., percent correct and Levenshtein Distance). In this study, we analyse the data from these two tasks only, as they are the only ones pertaining to serial memory of landmarks. The extent to which route learning is supported/reliant upon sequence learning is beyond the scope of the present study, but that dataset does provide an opportunity to explore characteristics of sequence learning in a naturalistic wayfinding environment that hitherto has only received limited research. Indeed, previous studies have focussed on self-reported recall of navigation experience (Helstrup & Magnussen, 2001; Stawarczyk & D’Argembeau, 2019) or directional knowledge (Cornell et al., 1996; Meilinger et al., 2016), which represent only a limited portion of overall spatial representations (Chrastil, 2013). Given the important role that landmarks at intersections play in the successful navigation of routes (Janzen, 2006; Janzen & Weststeijn, 2007; Waller & Lippa, 2007), the present study is the next step in broadening our understanding of serial position memory in a realistic navigation scenario.

Distinct analysis of the different sequence learning measures is important as methodological differences between tasks have been shown to qualitatively affect the serial position function (e.g., Guérard & Tremblay, 2008; Ward et al., 2005). Indeed, those aforementioned studies have shown that when the same task demands were applied to different stimulus types, the serial position functions were qualitatively equivalent (instead, it is changes to the task that qualitatively affects the shape of the curve). If the serial position is task (rather than stimuli) dependent (Ward et al., 2005), then one might predict that our post-route learning versions of IFR and free RoO tasks might exhibit behavioural similarities to their respective conventional single learning trial versions of the tasks.

In IFR, participants are required to recall the preceding sequence in any order. This task typically produces strong recency and some primacy (e.g., Grenfell-Essam et al., 2013; Grenfell-Essam & Ward, 2012; Murdock, 1962; Ward et al., 2010), with this function shown across a range of stimulus types (Cortis et al., 2015; Spurgeon et al., 2014). While previous studies have shown serial position effects for the free recall of both intersection decision points (Cornell et al., 1996; Meilinger et al., 2016) and vividness ratings for landmarks along a familiar route (Helstrup & Magnussen, 2001), we examine the pattern of recall accuracy for the landmarks encountered along the route.

In addition to examining serial position functions, we perform further analysis on the IFR data by investigating output order (indeed, the effect of output order on the serial position function is one of the benchmark findings of short-term memory, Oberauer et al., 2018). Recall of shorter lists tends to be initiated with early list items, whereas recall of longer lists is often initiated with latter list items (e.g., Cortis et al., 2015; Grenfell-Essam et al., 2013; Grenfell-Essam & Ward, 2012; Spurgeon et al., 2014; Ward et al., 2010). Given that our route contains 12 landmarks (a relatively long list, for example, Ward et al., 2010), we might predict that participants would opt to initiate recall with latter list items and therefore exhibit a strong recency effect.

Furthermore, we investigate lag functions for the order of recall in IFR. Lag refers to response transitions for each successive pair of items (i.e., the lag in transition from the position of the first and second items in the recalled pair, where a lag of +1 indicates recall of successive items in the original sequence). The frequency of lags is assessed via conditionalised response probabilities (CRP; see Kahana et al., 2008; Ward et al., 2010) for which the frequency each lag occurs during recall is conditionalised on the number of chances to make that lag. In IFR, lag analysis typically reveals higher probabilities for smaller positive lags (with lag +1 indicating successive recall of items; Kahana, 1996; Kahana & Howard, 2005), which evidences chaining during recall. Chaining is thought to reflect a contiguity effect where recall of one item triggers the recall of proximal list items (for a review see Healey et al., 2019). Thus, if memory for landmarks along a route is organised according to how they relate with nearby landmarks, then we would expect high CRPs for lower lags.

In RoO, participants are re-presented with the list items and are required to identify the order of original presentation. This task has been shown to produce strong primacy and some recency across a range of stimulus types (e.g., Avons, 1998; Guérard & Tremblay, 2008; Johnson et al., 2016; Parmentier & Jones, 2000; Ward et al., 2005). However, the present study used RoO free, where output order is unconstrained. This task has also been shown to produce a serial position function exhibiting primacy and recency (e.g., Lewandowsky et al., 2008, 2009; Neath, 1997; although recency was stronger for longer lists, see Ward et al., 2010). The present study therefore examines whether canonical serial position effects are found for the order memory of landmarks encountered along a route.

RoO can also be assessed using lag CRPs. One benefit of examining sequence CRPs is that they provide a measure of relative order memory, as opposed to serial position functions which examine absolute order knowledge. The resultant CRP-lag function typically exhibits a peak at +1 lag, with an asymmetric lag recency effect illustrating more transitions for adjacent positions (i.e., transpositions close to the correct position are more frequent indicating some vague yet inexact positional knowledge) and a greater tendency to transition forward. Of specific relevance to the current study is that such temporal contiguity effects (i.e., the tendency to output successive items at test that were positioned nearby during encoding) have been found with delayed testing (Howard et al., 2008), prolonged learning (Cortis Mack et al., 2017), and when those long-term memories are for autobiographical events (Moreton & Ward, 2010). These findings indicate that temporal contiguity may also be a universal feature of sequence memory.

We further analysed the free RoO data via transposition CRPs, which refers to the distance of each landmark from its absolute serial position. Whereas typical serial position curves are concerned with simply correct or incorrect placements, analysis of transpositions indicates whether errors are nonetheless close to the correct position. We expected that transposition and lag analyses would be similar in their outcome, as they both assess the extent to which items are ordered, but the two measures provide a distinct view of absolute and relative positional knowledge (see Schoo et al., 2014 on the importance of distinguishing relative and absolute measures of order memory).

Another advantage of our reanalysis of Hilton et al. (2021) is that it enables a comparison across age groups. In the original study, older adults took longer to learn the route to criterion, but once at 90%, did not differ from the younger adults in respect to both free recall and associative learning. Older adults were, however, significantly poorer at free RoO in terms of correct absolute placement. This is consistent with previous ageing studies in which participants also completed free RoO for landmarks previously encountered along a route through a virtual environment, with older adults producing smaller correlations between their given sequence and the correct sequence (Allison & Head, 2017; Head & Isom, 2010). As in Hilton et al. (2021), these studies contained no analysis of serial position functions, and thus only revealed a quantitative reduction in sequence knowledge of the older adults.

Our further analysis allows us to explore whether these differences are merely quantitative or reflect qualitative differences in sequence memory. Conventional single-trial measures of sequence memory for older adults show broadly qualitatively equivalent functions for item (Kahana et al., 2002; Korsnes & Magnussen, 1996; Ward & Maylor, 2005) and order memory (e.g., Maylor et al., 1999), despite overall lower accuracy levels. Lower IFR levels in older adults have been linked to both reduced rehearsal (Ward & Maylor, 2005) and reduced forward ordered recall (Kahana et al., 2002). These findings suggest that any behavioural sequence memory effects reported for older adults in the present study would differ quantitatively to that shown with younger adults but not qualitatively.

However, the extent to which findings from the conventional single learning trial paradigms generalise to the present procedure is unclear, with three important methodological distinctions. First, the current procedure involves a single testing trial for a sequence following multiple exposures to the to-be-remembered sequence (i.e., the route). It is possible that multiple exposures to the to-be-remembered sequence might qualitatively change behaviour. Moreover, in respect to age differences, Griffin et al. (2017) reported older adults acquired less information across multiple learning trials compared to younger adults, although this decrement was linked to poor initial recall on the first exposure. The Hebb (1961) repetition procedure is another task in which participants receive multiple exposures to the same sequence (albeit surreptitiously). Despite multiple exposures, participants still exhibit the canonical serial position functions shown in single trial learning (e.g., Horton et al., 2008; although it is worth noting that older adults show impaired Hebb repetition effects for visuo-spatial stimuli, Turcotte et al., 2005).

A second important methodological difference is that participants were not instructed to learn the sequence of landmarks. However, while it remains unclear as to what extent route learning is underpinned by sequence learning, we argue that this should not affect the demonstration of established serial position effects for either IFR or free RoO. This is because even if landmark order is inconsequential to route learning, implicit memories have been shown to produce primacy and recency (e.g., Raanaas & Magnussen, 2006; see also Stawarczyk & D’Argembeau, 2019, where participants were not explicitly instructed to remember their thoughts). That said, encoding of landmark order is of route learning utility as forthcoming navigational decisions are primed, thus improving the efficiency of navigation (Schinazi & Epstein, 2010). Moreover, without any sequence knowledge, it would be difficult, if not impossible, to distinguish situations that feature similar or identical landmarks (Strickrodt et al., 2015).

The third difference is that the current task does not involve immediate retrieval of the sequence. However, as noted above, memory advantages for the boundary items in lists are a universal feature of sequence memory (Kelley et al., 2015). Indeed, long-term sequence learning tasks have reported recency effects suggesting that recency is not reliant upon short-term memory (Baddeley & Hitch, 1977; Pinto & Baddeley, 1991; see also Bhatarah et al., 2006). However, more recently, Cortis Mack et al. (2017) provided limited evidence for time-invariant serial position effects. They examined sequence memory following the presentation of list items over long intervals (one word every hour via a smartphone). While relatively shallow serial position functions were reported, there were strong temporal contiguity effects. It is, however, worth noting that the curvature of functions was more pronounced following analysis of the first trial only (a single trial reanalysis more in line with the present methodology and that of Baddeley & Hitch, 1977 and Pinto & Baddeley, 1991).

The overall aim of the present study was to investigate the presence of standard serial position effects in a realistic navigation task which has substantial methodological differences to typical sequence learning paradigms. To achieve this aim, we apply an array of analyses commonly used in serial learning paradigms to data for landmark memory and sequence knowledge for the first time. Taken together, existing studies suggest that benchmark sequence position effects should be observed following the present methodology despite the use of a single trial with multiple list exposure. Specifically, our key predictions were that both IFR and free RoO should exhibit the canonical serial position curves with both primacy and recency. For IFR, we predicted a tendency to initiate output with later list landmarks, thus accentuating the recency effect. We expected higher probabilities of smaller lags for both IFR and free RoO, demonstrating a greater likelihood of subsequent recall for landmarks positionally adjacent in the original sequence to the recalled item. For free RoO we expected a similar pattern in the transposition errors, with smaller transpositions indicating that even incorrectly placed landmarks occur somewhat close to their actual position. With respect to the role of age, we predict quantitative reduction but not qualitative differences in the pattern of sequence memory for older, relative to younger, adults.

## Method

In this study, we performed additional analyses on the data collected by Hilton et al. (2021). That study comprised three experiments each beginning with a route learning phase. Experiment 1 involved a “Fixed Learning” protocol (3 exposures of the route), whereas Experiments 2 and 3 employed a “Flexible Learning” protocol wherein participants were trained to criterion (90%). In Flexible Learning, participants were exposed repeatedly to the route until they gave 90% of the directions correctly, at which point the participants received no more exposures to the route and moved onto the test phase. The inclusion of the Missing Landmark Task in Experiment 3 of the original study is its only procedural distinction from Experiment 2. As we do not analyse that task in the present study, Experiments 2 and 3 were combined into one Flexible Learning condition for increased statistical power and parsimonious analysis. We have not included a detailed description of the Associative Cue and Missing Landmark tasks in the present study as they are not addressed and are not critical to the study design required to produce the data we analyse.

### Participants

In the Fixed Learning condition, there were 29 younger and 27 older participants. In the Flexible Learning condition, there were 59 younger and 50 older participants. Older participants were screened for mild cognitive impairment using the Montreal Cognitive Assessment (MoCA; Nasreddine et al., 2005). All older participants scored above the MoCA cut-off score of 23 (Luis et al., 2009; Waldron-Perrine & Axelrod, 2012). See Table 1 for participant information. Ethical approval was granted by the Bournemouth University Research Ethics Panel and written informed consent was gained from all participants who either received course credits or an honorarium.

**Table 1.** Participant information.

	Sex	*n*	Age			MoCA	
			Mean	*SD*	Range	Mean	*SD*
Fixed learning condition							
Younger	Female	16	22.38	4.84	18–35		
	Male	13	19.69	1.11	18–22		
Older	Female	14	71.14	5.76	64–82	26.35	2.06
	Male	13	70.77	3.39	65–77	26.08	2.22
Flexible learning condition							
Younger	Female	30	22.00	3.70	18–35		
	Male	29	22.97	4.46	18–33		
Older	Female	27	71.04	4.79	66–86	27.00	2.11
	Male	23	71.83	6.08	65–90	26.74	2.05

*SD*: standard deviation.

### Design

A three-factor (2 × 2 × 12) mixed multifactorial design was employed. The between groups independent variables were age group (2 levels: younger and older) and learning condition (2 levels: Fixed Learning and Flexible Learning), and the within groups variable was serial position (1–12). The two dependent measures were serial recall accuracy for IFR and Free RoO.

### Learning phase

**Figure 1.**
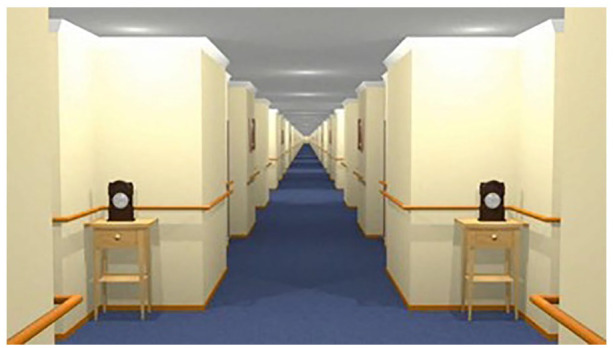
A screenshot of an intersection in the environment.

Participants were instructed to learn a route through a virtual environment. The route consisted of 12 intersections (4 left turns, 4 right turns, and 4 straight ahead). Each intersection had a pair of identical landmarks. The landmarks at each intersection were unique from all other intersections and only one pair of landmarks could be seen at a time (see Figure 1). The order of landmarks and route directions were randomised for every participant. They were shown videos of passive transportation along the route. At each intersection, the footage was paused and participants were required to indicate the direction of travel (right, left, straight) required to continue along the route. Transportation resumed once a response was given thus providing immediate feedback.

In the Fixed Learning condition, participants navigated the route three times during the Learning Phase. Participants in the Flexible Learning condition navigated the route repeatedly until they reached a 90% performance criterion (i.e., they responded correctly at 11 out of the 12 intersections). Once participants navigated the route with at least 90% correct responses, the Learning Phase was terminated. In the Flexible Learning condition, younger adults took an average of 3.71 attempts to pass the learning phase and older adults took an average of 5.26 attempts.

### IFR

Participants were asked to verbally free recall as many of the landmarks from the route as they could remember (i.e., recall the list in any order). Any ambiguous responses were clarified with the participant by asking for alternative names and visual descriptions of the object. Responses were recorded by the experimenter in the order they were output by the participant.

### Free RoO

Following IFR, participants were presented with printed images of all the landmarks from the route and were required to arrange them into the order in which they occurred along the route. Participants were able to place landmarks into their positions in any temporal order (i.e., output order was unconstrained) and were free to change their decisions before finalising the order. The sequence was recorded once participants indicated reconstruction was complete.

### Procedure

Participants completed the Learning Phase and were not informed about the requirements of the forthcoming tasks to avoid participants intentionally adapting their learning strategy. Thus, participants did not know that the identity or sequence of landmarks would be tested. After the Learning Phase, participants completed the IFR task and then the Free RoO task. As previously mentioned, participants also completed two other tasks which are not discussed in this study but are summarised in the introduction section. The order of the tasks in the test phase was counterbalanced, with the proviso that the first test was always IFR, to prevent additional learning of landmark identities from the other test tasks.

### Data analysis

We analysed the data using linear (LME) and generalised linear mixed effect models (GLME) in *R* (R Core Team, 2019) using the lme4 package (version 1.1-21; Bates et al., 2015). The lmerTest package (version 3.1-3; Kuznetsova et al., 2017) was used to estimate *p* values for LME models using Satterthwaite’s method. Due to the low number of observations per participant, we used intercept only random effects structures to preserve statistical power. For all models, we included participant and landmark identity as random factors. Due to issues with model convergence, data from flexible learning and the fixed learning groups were analysed separately. Models from the flexible learning condition additionally included the number of repetitions as a random effect to account for variations in route exposure in the learning phase.

## Results

### IFR task

#### Serial position memory

Responses from the IFR task were scored as described in Ward et al. (2010), with items being assigned a 1 if they were recalled and a 0 if they were not recalled.

We ran a GLME model separately for the fixed learning and flexible learning conditions with the outcome variable as recall probability (0 or 1). Landmark position was included as an ordered factor with polynomial contrast coding to identify trends within the data, and age group was included as a fixed effect (younger or older). Estimates, standard errors, *z*-values, and *p* values are reported in Table 2. There was no significant effect of age group on recall proportions in either condition.

**Table 2.** Coefficients from the fixed learning and flexible learning IFR serial position GLME analysis.

Fixed effect on recall probability	Fixed learning model				Flexible learning model			
	Estimate	Std. error	*z*-value	*p* value	Estimate	Std. error	*z*-value	*p* value
Intercept	**0.54**	**0.21**	**2.59**	.009*	**0.70**	**0.17**	**4.01**	<.001*
Age group	0.19	0.14	1.34	.179	0.07	0.11	0.60	.549
Linear fit—serial position	**2.35**	**0.35**	**6.72**	<.001*	0.34	0.22	1.52	.129
Quadratic fit—serial position	**0.72**	**0.33**	**2.17**	.030*	0.34	0.22	1.55	.122
Cubic fit—serial position	0.34	0.33	1.03	.302	<0.01	0.22	0.01	.992
Age group × Linear fit	0.66	0.34	1.93	.054	0.27	0.22	1.23	.220
Age group × Quadratic fit	0.37	0.33	1.12	.263	–0.40	0.22	–1.82	.069
Age group × Cubic fit	**–0.74**	**0.33**	**–2.27**	.023*	–0.06	0.22	–0.28	.781

IFR: Immediate Free Recall; GLME: generalised linear mixed effect.

* Significant *p* values (|*p*| < .05) in bold.

**Figure 2.**
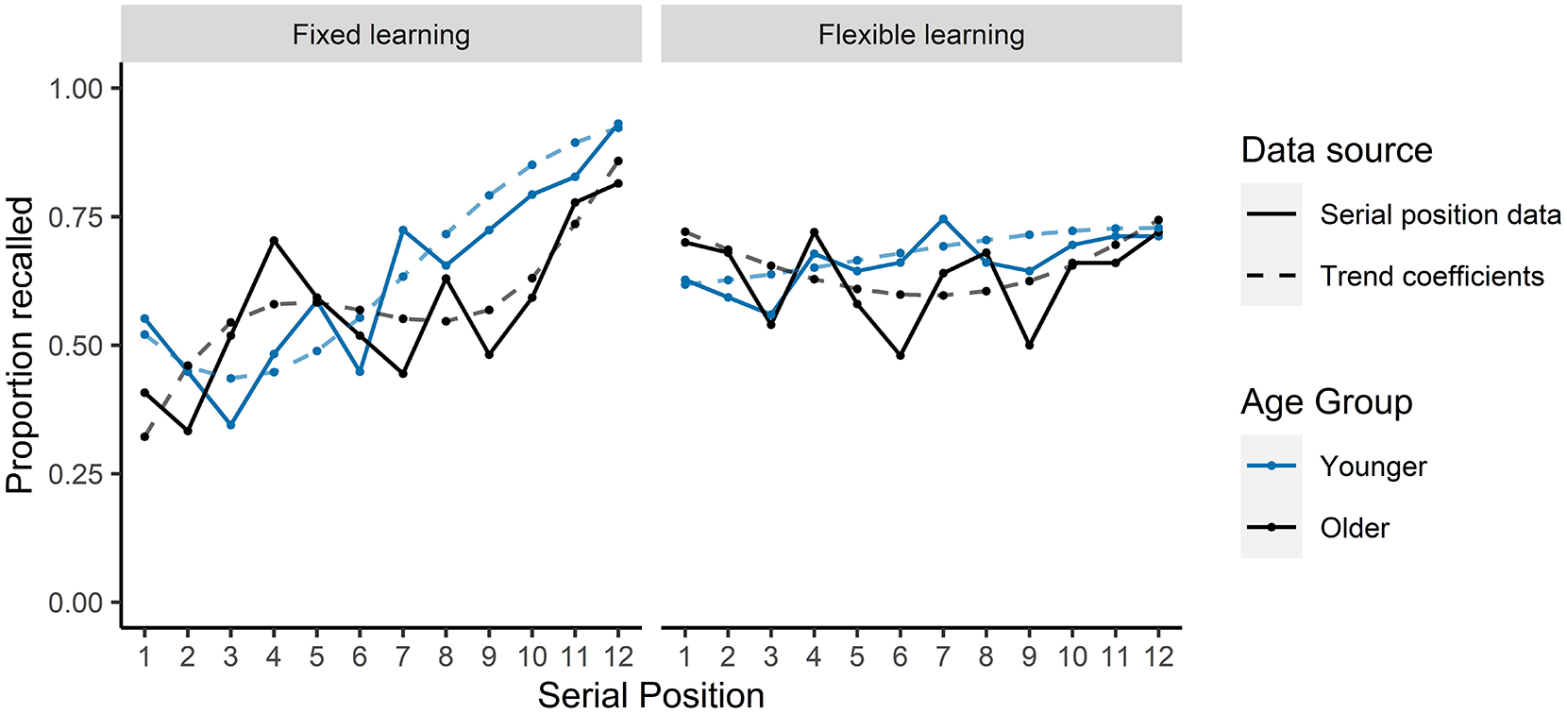
Mean proportion of words recalled in the IFR task as a function of serial position and trend effects from GLME models.

For the fixed learning condition, recall of landmarks as a function of serial position was best described by a linear trend and this did not interact with age. There was an age group by cubic fit interaction which suggests that recall proportions of older adults could be described by a cubic fit better than that of the younger adults. However, this interaction with a cubic fit (β = −0.74) was weaker than the overall linear fit (β = 2.35). Overall, there was a linear effect of serial position on landmark recall probability for both older and younger age groups (see Figure 2), for which an accuracy benefit was observed for latter route landmarks. For the flexible learning condition, there was no significant fit of any trend to the recall proportions as a function of serial position and no interactions with age. This suggests that there was no effect of serial position on recall probability in the flexible learning condition (see Figure 2).

This analysis indicates that in the fixed learning condition, there was a recency effect on recall such that landmarks at the end of the route were more likely to be recalled than items in earlier positions along the route. In contrast, no trend was observed on recall in the flexible learning condition which suggests that serial position of landmarks along the route did not affect likelihood of that landmark being recalled.

#### Order of output

**Figure 3.**
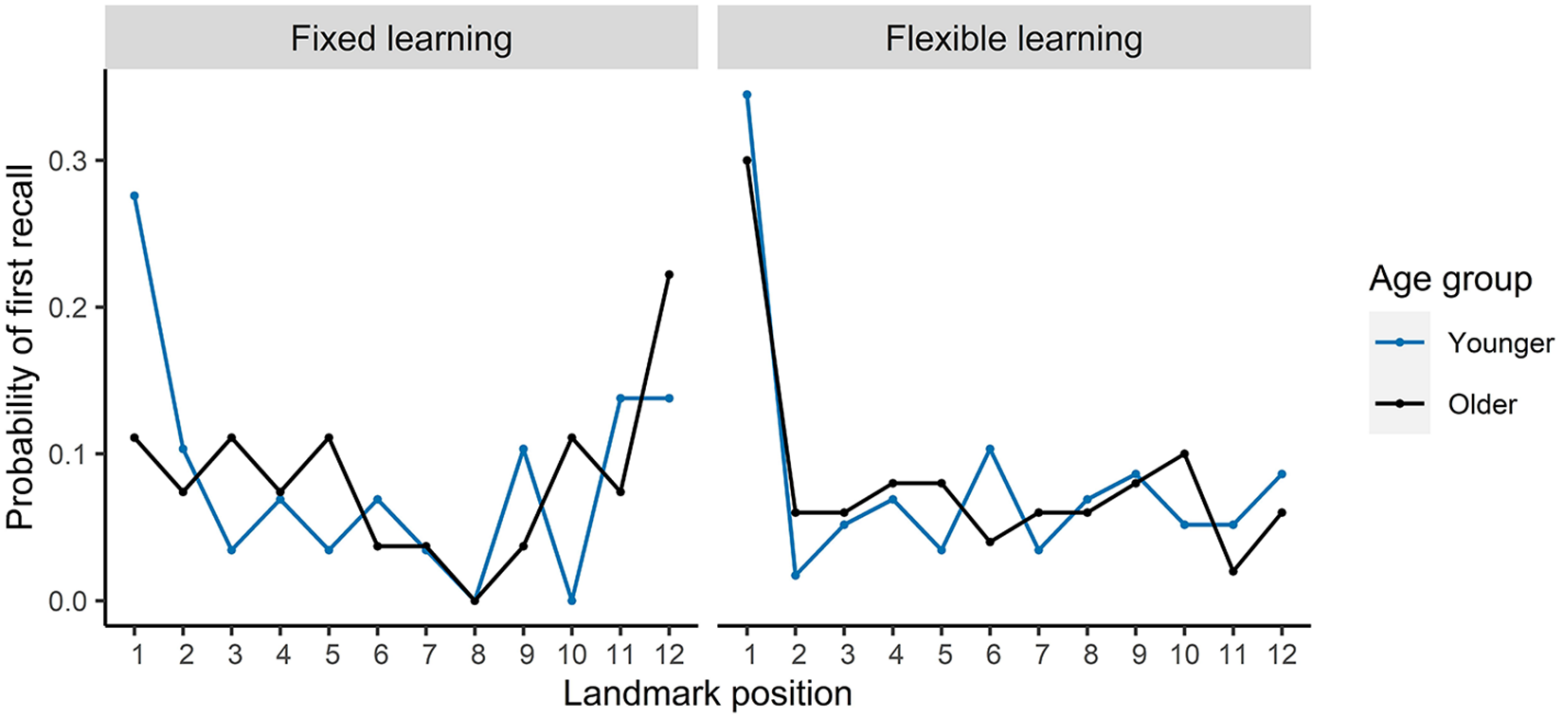
Probability of first recall for landmarks in each serial position.

To examine potential primacy or recency effects in recall strategy, Figure 3 displays the probability of first recall (PFR) for each landmark position. PFR refers to probability that the initial item recalled was located in each of the serial positions during learning. For the fixed learning condition, younger adults showed a clear primacy effect which was not present for the older adults. In contrast, the older participants showed evidence of a recency recall strategy. There was some evidence of a recency effect in the younger participants in the fixed learning condition also, with the final 2 items having higher PFR than items 2–10; however, this recency peak was not as large as that of the older participants. For the flexible learning condition, the older participants showed a marked shift from recency towards primacy, compared with the older participant sample in the fixed learning condition. This tendency towards primacy in first recall was also present for the younger participants in the flexible learning condition. In fact, the reduction in recency effect was sharp for both age groups, with the final items in the flexible learning condition having equal PFR to all other items excluding the first.

#### Lag conditionalised response probabilities

The scoring method for the serial position curves assesses absolute positional knowledge but is insensitive to relative order. That is, a participant may place items in the incorrect absolute position during reconstruction, but still place items in the correct order relative to the last retrieved item. To analyse relative ordering of items, we computed conditionalised response probabilities (CRPs) at different lags (e.g., Kahana et al., 2008; Ward et al., 2010). Lag refers to the distance between each successive item in the given sequence in terms of their serial position during learning (e.g., recalling items 3 and 7 next to each other would produce a lag of 4). A lag may be negative if an item is recalled before an item with a lower serial position (e.g., recalling item 7, then 3 would result in a lag of −4). The CRP refers to the probability that each lag is made within a recalled list, after controlling for the number of opportunities available for each lag distance (for example, a lag of 11 can only occur once in a list of 12 items, whereas there are 10 opportunities to make a lag transition of 2).

**Figure 4.**
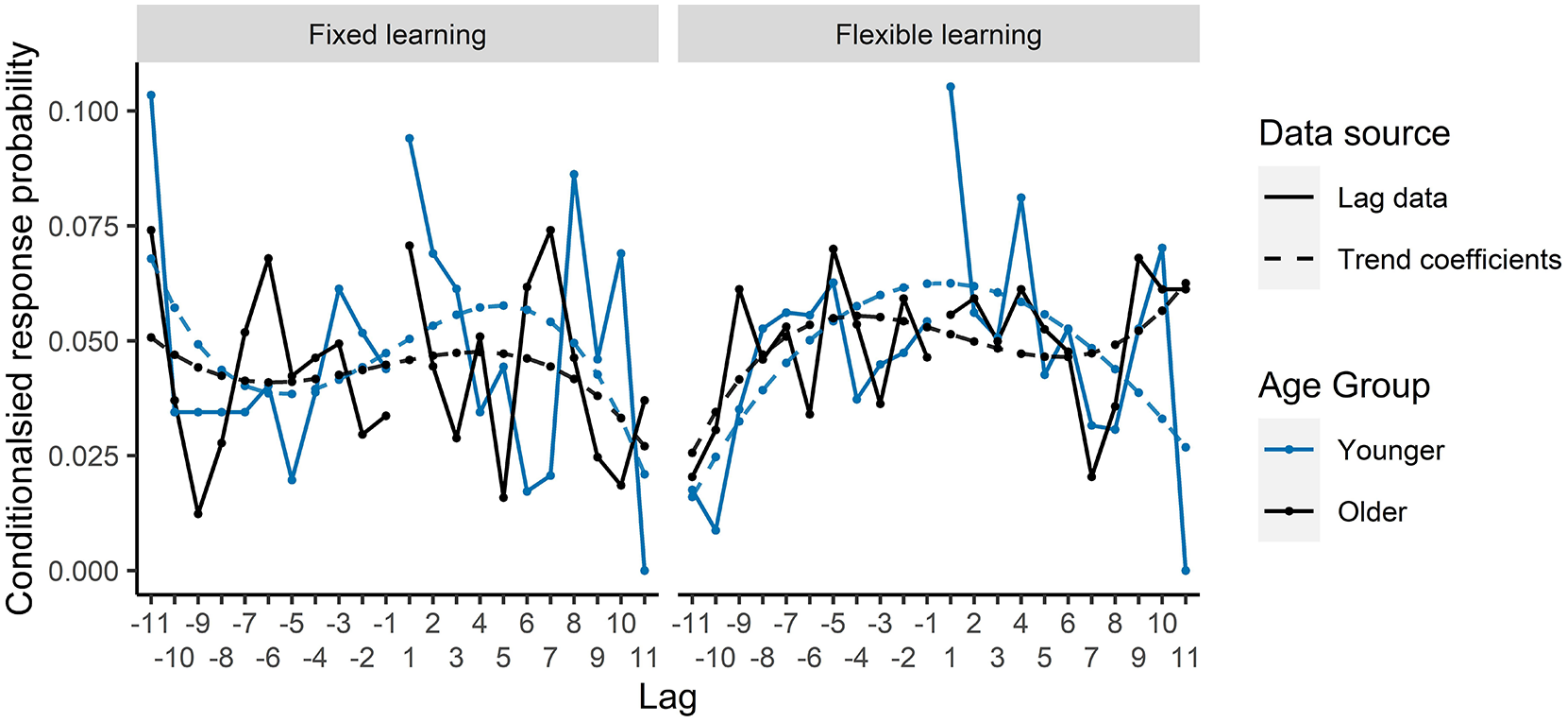
Lag-CRP curves and trend effects for each condition for the IFR task.

We ran LME models separately for the fixed learning and flexible learning conditions with the outcome variable lag CRPs. Lag was included as a factor with polynomial contrast coding to identify trends within the data, and age group (younger or older) was included as a fixed effect using sum contrast coding. Estimates, standard errors, *t*-values, and *p* values are reported in Table 3. There was a significant fit of lag CRP to a cubic trend in fixed learning, with no interactions between trend fits and age. Specifically, both age groups made more positive lags, indicating forward recall of landmarks. In the flexible learning condition, there was a significant fit of lag CRP to a quadratic trend, which interacted with age such that the fit was stronger for the younger age group (see Figure 4). This inverted U shape for the younger participants, peaking at lag +1, shows a bias towards lags of smaller values, revealing relative chaining of landmarks based on their serial order in IFR for the younger participants, but not older.

**Table 3.** Coefficients from the IFR CRP lag LME analysis.

Fixed effect on lag CRP	Fixed learning model				Flexible learning model			
	Estimate	Std. error	*t*-value	*p* value	Estimate	Std. error	*t*-value	*p* value
Intercept	**0.05**	**<0.01**	**14.26**	<.001*	**0.05**	**<0.01**	**22.32**	<.001*
Age group	<0.01	<0.01	0.67	.500	<–0.01	<0.01	–0.39	.693
Linear fit—lag	–0.01	0.02	–0.76	.451	0.02	0.01	1.45	.149
Quadratic fit—lag	<–0.01	0.02	–0.56	.575	**–0.04**	**0.01**	**–3.70**	<.001*
Cubic fit—lag	**–0.03**	**0.02**	**–2.15**	.032*	<0.02	0.01	1.55	.123
Age group × Linear fit	<–0.01	0.02	–0.02	.983	<–0.01	0.01	–0.37	.710
Age group × Quadratic fit	<0.01	0.02	0.10	.920	**–0.03**	**0.01**	**–2.48**	.013*
Age group × Cubic fit	–0.01	0.02	–0.90	.371	–0.01	0.01	–1.19	.234

IFR: Immediate Free Recall; CRP: conditionalised response probabilities; LME: linear mixed effect.

* Significant *p* values (|*p*| < .05) in bold.

### Free RoO task

#### Serial position memory

Responses from the Free RoO task were scored as described in Ward et al. (2010), with items being assigned a 1 if they were placed in the correct position in the sequence and a 0 if they were transpositions.

We ran a GLME model separately for the fixed learning and flexible learning conditions with the outcome variable as performance (0 or 1). Landmark position was included as an ordered factor with polynomial contrast coding to identify trends within the data, and age group was included as a fixed effect (younger or older). Estimates, standard errors, *z*-values, and *p* values are reported in Table 4.

**Table 4.** Coefficients from the fixed learning and flexible learning free RoO serial position GLME analysis.

Fixed effect on recall probability	Fixed learning model				Flexible learning model^a^			
	Estimate	Std. error	*z* value	*p* value	Estimate	Std. error	*z*-value	*p* value
Intercept	**–0.74**	**0.18**	**–4.11**	<.001*	**–0.52**	**0.13**	**–4.01**	<.001*
Age group	**0.65**	**0.16**	**3.96**	<.001*	**0.58**	**0.13**	**4.55**	<.001*
Linear fit—serial position	**–1.83**	**0.34**	**–5.43**	<.001*	**–1.71**	**0.24**	**–7.09**	<.001*
Quadratic fit—serial position	**2.99**	**0.36**	**8.27**	<.001*	**3.21**	**0.26**	**12.21**	<.001*
Cubic fit—serial position	0.44	0.34	1.30	.194	–	–	–	–
Age group × Linear fit	0.05	0.33	0.16	.870	0.23	0.24	0.97	.330
Age group × Quadratic fit	0.23	0.25	0.67	.503	0.13	0.25	0.51	.610
Age group × Cubic fit	0.03	0.34	0.08	.936	–	–	–	–

RoO: Reconstruction of Order; GLME: generalised linear mixed effect.

a To achieve model convergence, polynomial contrasts were run to identify linear and quadratic trends only.

* Significant *p* values (|*p*| < .05) in bold.

**Figure 5.**
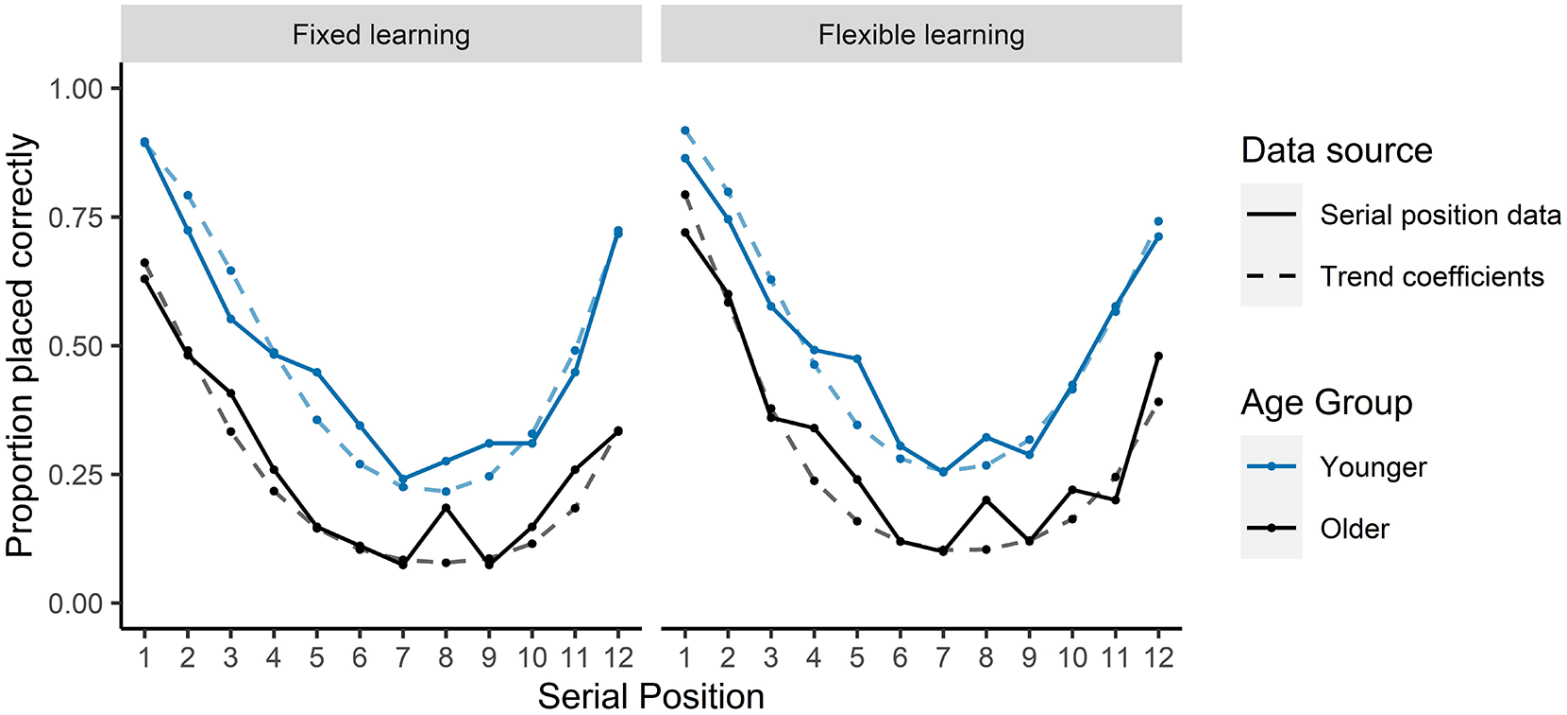
Mean proportion of landmarks placed correctly in the Free RoO task as a function of serial position and trend effects from GLME models.

For both conditions, there was a significant effect of age such that younger participants performed better than older participants. Both linear and quadratic trends provided a significant fit to the data. The fit of a quadratic trend was stronger than a linear trend in both fixed learning and flexible learning conditions. There were no interactions between trend fits and age group. Overall, there was a quadratic effect of serial position on probability of correct landmark placement (see Figure 5). This trend demonstrates primacy and recency benefit in serial order memory for both age groups and across fixed and flexible learning protocols.

#### Lag conditionalised response probabilities

**Figure 6.**
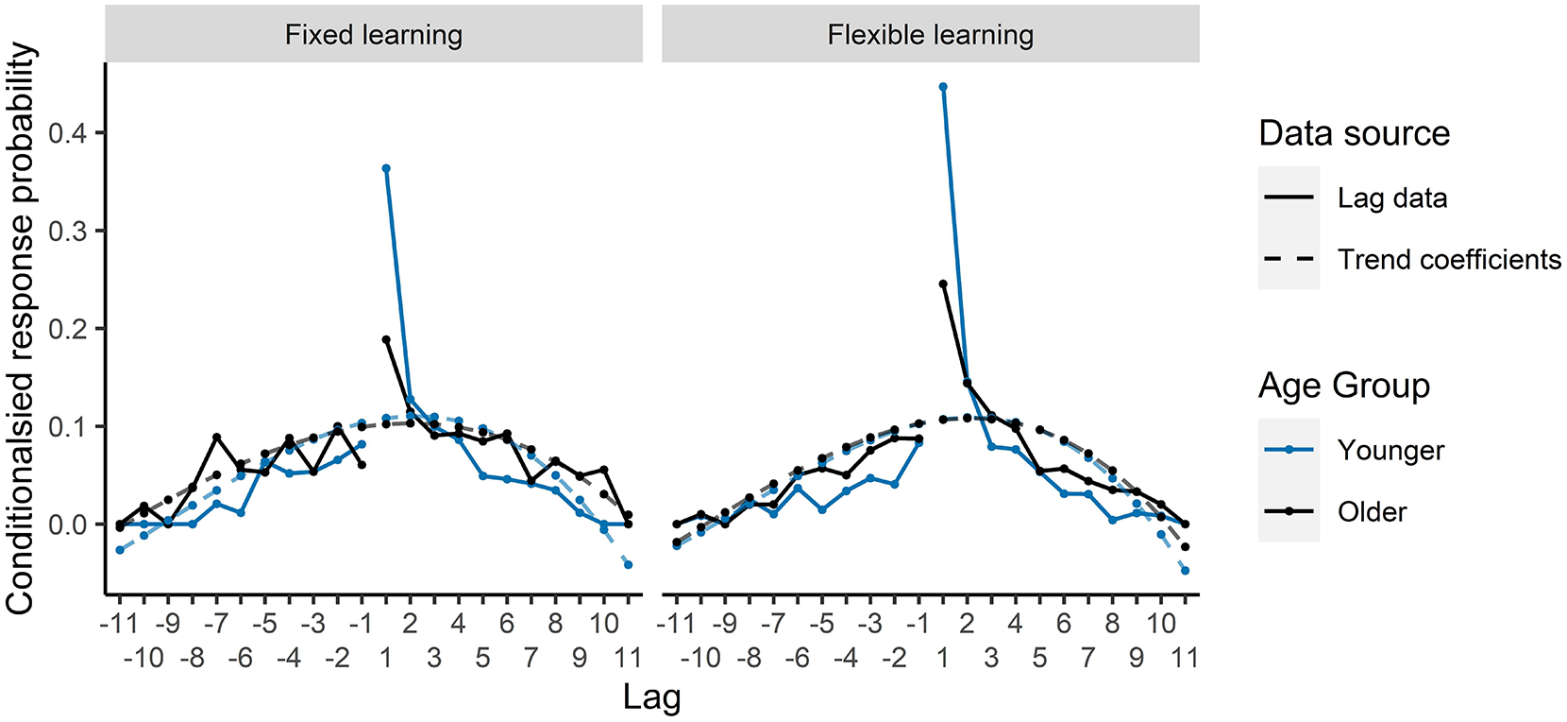
Lag-CRP curves and trend effects for each condition for the Free RoO task.

We ran LME models separately for the fixed learning and flexible learning conditions with the outcome variable lag CRPs. Lag was included as a factor with polynomial contrast coding to identify trends within the data. Age group (younger or older) was included as fixed effects using sum contrast coding. Estimates, standard errors, *t*-values, and *p* values are reported in Table 5. There were significant fits of lag to linear, quadratic, and cubic trends, for which the fit of a quadratic trend was stronger than the fit of linear and cubic trends in both fixed learning and flexible learning conditions (see Figure 6). The fit of the quadratic trend interacted with age group such that the fit was slightly weaker for the older participants in the fixed learning condition, although this was still the best trend to describe their data overall. There was no interaction between age group and the quadratic fit in the flexible learning condition and no other significant interactions. This inverted U-shaped trend demonstrates a bias towards lags of smaller values, which shows that participants had good knowledge of the relative ordering of landmark sequence.

**Table 5.** Coefficients from the Free RoO CRP lag LME analysis.

Fixed effect on lag CRP	Fixed learning model				Flexible learning model			
	Estimate	Std. error	*t*-value	*p* value	Estimate	Std. error	*t*-value	*p* value
Intercept	**0.06**	**<0.01**	**19.24**	<.001*	**0.06**	**<0.01**	**25.56**	<.001*
Age group	–0.01	<0.01	–1.64	.102	<–0.01	<0.01	–1.15	.248
Linear fit—lag	**0.04**	**0.02**	**2.66**	.001*	**0.03**	**0.01**	**3.03**	.003*
Quadratic fit—lag	**–0.18**	**0.02**	**–12.41**	<.001*	**–0.20**	**0.01**	**–19.55**	<.001*
Cubic fit—lag	**–0.03**	**0.02**	**–2.27**	.024*	**–0.04**	**0.01**	**–4.18**	<.001*
Age group × Linear fit	<–0.01	0.02	–0.17	.864	<–0.01	0.01	–0.50	.617
Age group × Quadratic fit	**–0.03**	**0.02**	**–2.21**	.027*	–0.01	0.01	–1.03	.302
Age group × Cubic fit	–0.01	0.02	–0.98	.328	–0.01	0.01	–0.75	.455

RoO: Reconstruction of Order; CRP: conditionalised response probabilities; LME: linear mixed effect.

* Significant *p* values (|*p*| < .05) in bold.

From examination of Figure 6, the fit of the quadratic trend matched the data closely on almost all lag positions. However, there was a large departure of the data from the fitted trend for lag +1 across both conditions and age groups. This is not particularly surprising as a lag of +1 is special in that it reflects the correct relative placement of items in the sequence, while all other positions are lags in which participants made an error in the relative ordering. In a follow-up analysis, we analysed CRP to make +1 lags only. Cutting down the data to only examine CRP for +1 lag resulted in only one observation per participant, thus we used a linear model without a random effects structure. CRP for lag +1 was the outcome variable with fixed effects of age group (younger, older) and condition (fixed or flexible) both coded using sum contrasts. The model shows an effect of age group such that the probability of +1 lags was greater for younger participants than older participants (β = 0.09, *SE* = 0.02, *t* = 5.17, *p* < .001). There was no effect of condition (β = −0.04, *SE* = 0.02, *t* = −1.93, *p* = .056) and no significant interaction (β < −0.01, *SE* = .02, *t* = −0.36, *p* = .718). The model presented in Table 5 and Figure 6 shows that both age groups had a relative knowledge of the sequence above chance level; however, the model on +1 lags only suggests that this relative knowledge was finer grained for the younger participants than the older participants.

#### Transposition errors

Transposition error refers to the distance between a placed item in the sequence and its absolute correct position (as opposed to the lag analysis which quantifies the relative distance between adjacently placed items regardless of their overall position in the given sequence).

**Figure 7.**
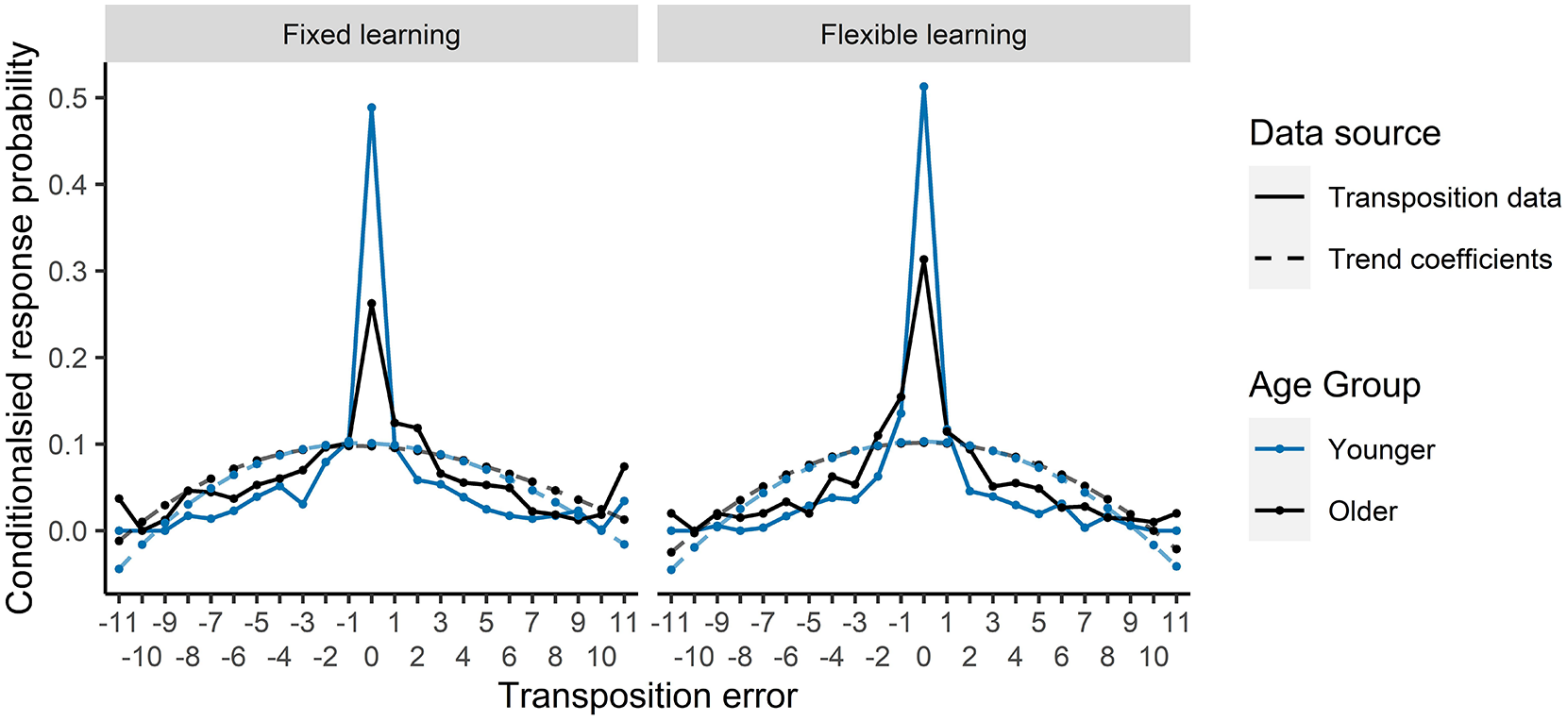
Transposition error curves and trend effects for each condition for the Free RoO task.

We ran LME models separately for the fixed learning and flexible learning conditions with the outcome variable transposition error CRPs. Transposition error was included as a factor with polynomial contrast coding to identify trends within the data. Age group (younger or older) was included as fixed effects using sum contrast coding. Estimates, standard errors, *t*-values, and *p*-values are reported in Table 6. There were significant fits of lag to a quadratic trend in both fixed learning and flexible learning conditions that did not interact with age group (see Figure 7). This inverted U-shaped trend demonstrates a bias towards smaller transposition errors, which shows that even when errors were made, they were close to the correct position. A difference between age groups can be visually identified at transposition 0 for both conditions in Figure 7. This difference is analogous to the main effect of age we reported in the serial position analysis for Free RoO (Table 4), showing overall better placement of items in their correct positions for younger adults.

**Table 6.** Coefficients from the fixed learning and flexible learning free RoO transposition error LME analysis.

Fixed effect on lag CRP	Fixed learning model				Flexible learning model			
	Estimate	Std. error	*t*-value	*p* value	Estimate	Std. error	*t*-value	*p* value
Intercept	**0.06**	**<0.01**	**16.99**	<.001*	**0.05**	**<0.01**	**22.77**	<.001*
Age group	<–0.01	<0.01	–1.32	.190	<–0.01	<0.01	–1.39	.160
Linear fit—lag	0.01	0.02	0.75	.460	<0.01	0.01	0.26	.790
Quadratic fit—lag	**–0.12**	**0.02**	**–10.87**	<.001*	**–0.21**	**0.01**	**–18.81**	<.001*
Cubic fit—lag	0.02	0.02	1.31	.190	<0.01	0.01	0.18	.860
Age group × Linear fit	<0.01	0.02	0.12	.910	<0.01	0.01	0.01	.990
Age group × Quadratic fit	–0.03	0.02	–1.62	.110	–0.02	0.01	–1.49	.140
Age group × Cubic fit	<0.01	0.02	0.03	.970	<0.01	0.01	<0.01	.999

RoO: Reconstruction of Order; CRP: conditionalised response probabilities; LME: linear mixed effect.

* Significant *p* values (|*p*| < .05) in bold.

## Discussion

The present study provides a detailed examination of landmark sequence memory in a route learning task. Here, we re-analyse data from Hilton et al. (2021) and demonstrate some of the classical sequence learning effects. We tested sequence learning via IFR and Free Reconstruction of Order (Free RoO) for the 12 landmarks encountered at decision points along a route. Following both fixed and flexible learning, Free RoO produced the canonical bowed serial position effects found in conventional list learning tasks (e.g., Lewandowsky et al., 2008, 2009; Neath, 1997; Tan & Ward, 2008; Ward et al., 2010). Established patterns of sequence learning were seen also in the lag and transposition CRP functions which revealed an asymmetric lag recency. IFR of landmarks produced serial position functions that were, however, less consistent with earlier findings. For fixed learning, there was evidence of a recall benefit for latter list items, whereas the flexible learning condition produced much flatter functions. These functions were at odds with the order of output for the free recall of landmarks, which revealed a bias towards outputting early list items first. Little evidence for contiguity in recall was found via lag CRP analysis, although some evidence for forward recall emerged for the younger adults following flexible, but not fixed learning. The only other main effect of age was found only with overall Free RoO scores, but did not affect the bowed serial position trend.

The serial position function exhibited in the Free RoO task demonstrates that participants did acquire knowledge for the order of landmarks in both the fixed and flexible learning conditions. The pattern of this serial position function is consistent with studies that have explored Free RoO for short-term memory of verbal sequences (e.g., Lewandowsky et al., 2008, 2009; Neath, 1997; Tan & Ward, 2008; Ward et al., 2010). Specifically, a memory advantage was observed for boundary items at both ends of the sequence, revealing both primacy and recency effects. This finding supports the notion that serial position effects for sequences extend beyond the standard list learning tasks and generalises to a navigation context. Such a finding is consistent with primacy and recency effects found in respect to both the increased memory vividness for landmarks positioned at the start and end of a frequently travelled route (Helstrup & Magnussen, 2001) and memory for thoughts encountered along a route (Stawarczyk & D’Argembeau, 2019).

Bowed serial position curves in Free RoO were observed for both older and younger age groups, despite an overall impairment for older adults. The presence of both primacy and recency was consistent with the serial position functions shown in previous studies with both younger and older samples (Elliott et al., 2011; Maylor et al., 1999; Surprenant, 2007). Specifically, the serial position function appears to differ quantitatively but not qualitatively for older adults (e.g., Kahana et al., 2002; Korsnes & Magnussen, 1996; Ward & Maylor, 2005). Moreover, an age-related impairment in contextual information (i.e., impaired recall of temporal location) is consistent with age-related memory deficits disproportionately affecting context (e.g., Kessels et al., 2007). This is known as the “associative deficit hypothesis” (e.g., Chalfonte & Johnson, 1996; Naveh-Benjamin, 2000), which posits that older adults are markedly impaired for bound/associative information. In the present study, we employed a surprise test of context (i.e., item-position association) and found an age-related deficit, consistent with the age-related deficit shown for a surprise test of spatial context (Lugtmeijer et al., 2019).

In contrast with the age-related deficits we report in Free RoO, we found no significant effect of age for IFR (see also Golomb et al., 2008). This is consistent with the proposition that contextual memory information is disproportionately affected by ageing (e.g., Kessels et al., 2007; although item-based deficits have been reported in older adults, e.g., Kahana et al., 2002; Ward & Maylor, 2005). Our present reanalysis contributes to that reported in Hilton et al. (2021) that the age-related route learning differences observed can be explained by specific impairments in sequence order memory. Hilton et al. (2021) showed that once rate of route learning was controlled (via flexible learning to criterion), older and younger adults differed only in reconstruction of landmark order (free RoO). The present re-analysis highlights that these differences are quantitative (rather than qualitative), with serial position and lag CRP functions qualitatively equivalent in Free RoO.

Lag functions for IFR showed no evidence of contiguity in the fixed learning condition, but evidence of forward contiguity for the younger adults emerged in the flexible learning condition more consistent with conventional lag effects in IFR (e.g., Ward et al., 2010). No such contiguity effects were observed for the older adults, consistent with previous research showing diminished free recall contiguity in ageing (Kahana et al., 2002). It is possible that greater exposure and learning of the route in the flexible learning condition led to forward recall strategies for the younger adults. However, this explanation is not supported by findings of strong lag functions in IFR for words presented over very short time scales (Ward et al., 2010).

As noted above, the IFR data were less consistent with established serial position effects than the Free RoO data, notably lacking the bowed serial position effects in recall of landmarks. For the fixed learning condition, there was evidence of recency (but not of primacy), whereas the flexible learning condition produced a relatively flat function. While stronger recency (compared to primacy) is consistent with free recall of longer lists (Grenfell-Essam et al., 2013; Grenfell-Essam & Ward, 2012; Spurgeon et al., 2014; Ward et al., 2010), this enhanced recency was accompanied by a tendency to initiate recall with latter list items. Analysis of output order in the present study revealed a bias towards outputting early list items first. Such a finding is inconsistent with the explanation that initiating recall with an item improves recall accuracy due to an absence of output interference (e.g., see Tan & Ward, 2008). That is, recall for the latter list items was superior despite recall being initiated with early list items. Such a trend contradicts a benchmark finding of short-term memory (Oberauer et al., 2018).

The lack of typical serial position effects in the IFR task cannot be attributed to the lack of serial memory in our participants, as those canonical curves are clearly present in the Free RoO task. Yet despite participants acquiring such sequence memory, it was not evident in free recall of items in the same way as in other sequence learning paradigms (e.g., Ward et al., 2010). One might argue it is unsurprising that some differences exist in our study given the vastly different task characteristics in the present study compared to typical sequence learning tasks. Indeed, Cortis Mack et al. (2017) did report weak serial position effects following free recall of a list presented over a prolonged (8 hr) duration despite reporting benchmark lag CRP functions. This finding suggests that free recall serial position functions may not be time invariant. Nevertheless, despite those task differences, the serial position functions are stark in the Free RoO task. It appears that the task differences did not affect the acquisition of serial order knowledge but did differentially affect how serial order memory was manifested in the IFR and Free RoO tasks. It is beyond the scope of the current study to provide a full framework for this phenomenon, but we discuss the possibilities here as avenues for future research.

One difference in our task compared to standard paradigms is the number of exposures to the sequence. In the present protocol, participants are presented with a single sequence to which they are exposed multiple times. This contrasts with the conventional protocols where participants respond following a single exposure to the sequence. Moreover, in the route learning task, both presentation of the sequence and the retention interval is considerably longer in duration than the conventional paradigms. Our study demonstrates that the bowed Free RoO function is resistant to longer intervals and multiple exposures to the list. Whereas the sensitivity of IFR to changes in list exposure is evident in the differences between the fixed and flexible learning conditions on both recall position and output order measures. The recency component is reduced for flexible learning relative to fixed learning (see Figure 2). Similarly, the extent to which participants initiate recall with the last item is reduced for flexible learning. It is not clear why flexible learning should result in a shift in recall strategy but the only difference between conditions is the number of exposures to the sequence (3 for fixed learning compared to a grand mean of 4.42 for flexible learning).

Given this shift towards a primacy-based output order, it is surprising that primacy is absent in the present free recall functions. Tan and Ward (2000) suggested that rehearsal of early list items, specifically the recency of that rehearsal, contributed to primacy. It is possible therefore that participants stopped rehearsing early list items in our study due to the lengthy presentation procedure (or did not engage in rehearsal at all). Indeed, interrupting rehearsal during list learning has been shown to eliminate primacy, but not recency serial position effects in recall (Marshall & Werder, 1972; see also Tan & Ward, 2000), which would explain the lack of primacy in both learning conditions. It is worth re-emphasising, however, that Cortis Mack et al. (2017) reported weak free recall serial position functions following prolonged sequence presentation despite some pronounced output order functions. The existence of recency in the fixed learning condition can be explained by the benefit of recency in output order which is not affected by the lack of rehearsal (Marshall & Werder, 1972; Tan & Ward, 2008).

Another methodological difference in our task is that participants were not explicitly instructed to learn the landmarks or their order in the route learning task. Notwithstanding this lack of instruction, we observed the serial position effect in Free RoO (consistent with the serial position functions for recall of thoughts experienced along a route, Stawarczyk & D’Argembeau, 2019). Indeed, the same landmarks are not repeated within a sequence, therefore, to learn the route participants could “simply” associate each landmark with a directional response (Waller & Lippa, 2007). Despite the non-essential nature of sequence information for the specific route learning task, participants acquired order memory as shown by both absolute (the Free RoO function) and relative (CRP-lag functions) measures of serial memory. The acquisition of sequence knowledge despite not knowing the forthcoming test is consistent with Tan and Ward (2007) who showed that pre-cueing the forthcoming reconstruction procedure (compared to post-cueing after the sequence has been presented) does not qualitatively affect the Free RoO serial position function. It is also unlikely that naivety of the upcoming tasks was responsible for the inconsistent IFR results, as null effects of task expectancy have previously been reported with IFR tasks (Bhatarah et al., 2008; Grenfell-Essam & Ward, 2012).

Given that the list was lengthy (12 landmarks) and presented over a prolonged period, it is conceivable that participants have segmented the list into smaller sub-lists. Horner et al. (2016) have shown that in navigating different virtual rooms, the spatial boundary (e.g., the doorway) functions to segment the sequence, with adjacent objects remembered better when within the same room rather than when positioned across adjoining rooms. It is possible that directional change during the route (i.e., turning left or right rather than continuing straight) could operate to segment the list. We were not able to leverage the present dataset to investigate this further as the sequence of turning directions along the route was randomised for each participant. It is therefore a question for future studies to examine whether turning directions can induce route segmentation. One might predict that segmentation would produce mini-serial position curves for each sub-list (where superior memory for the boundary items results from greater attentional focus, e.g., Faber et al., 2018) and reduced temporal contiguity across boundaries.

In summary, this study provides evidence of typical serial position memory effects for landmarks encountered during route navigation. The Free RoO task produced strong primacy and recency benefits for landmarks found at the beginning at the end of the route. This function existed for both age groups, despite an overall reduction in sequence knowledge for older adults. Interestingly, the serial position effects were not observed in IFR of landmarks which could be due to the several differences between our task and standard sequence learning tasks, although this avenue requires further empirical research. Despite these task differences, the serial position curves in the Free RoO task support the ubiquity of this function and the notion that primacy and recency are general properties of memory which extend to a navigation context.
